# The gait speed advantage of taller stature is lost with age

**DOI:** 10.1038/s41598-018-19882-1

**Published:** 2018-01-24

**Authors:** Alexis Elbaz, Fanny Artaud, Aline Dugravot, Christophe Tzourio, Archana Singh-Manoux

**Affiliations:** 1 Université Paris-Saclay, Univ. Paris-Sud, UVSQ, CESP, INSERM, Villejuif, France; 2 INSERM U897, Neuroepidemiology team, Bordeaux, France; 3 University of Bordeaux, Bordeaux, France; 4 Department of Epidemiology and Public Health, University College London, London, UK

## Abstract

Taller individuals walk faster but it is unknown whether this advantage persists at older ages. We examined the cross-sectional/longitudinal associations of height with gait speed (GS) in participants from the Dijon-Three-City cohort study (France) over 11 years. In 4011 participants (65–85 y), we measured usual/fast GS (6 m) up to five times. We examined whether the baseline height-GS association varied with age using linear regression, and whether height influenced GS change using linear mixed models. Taller participants 65 y at baseline walked faster than shorter ones (fast GS difference between top/bottom height quartiles, 0.100 m/s, *P* < 0.001); this association weakened with age (*P*-interaction = 0.02), with a 0.012 m/s (*P* = 0.57) difference at 80 y. Ten-year fast GS decline was 51% greater (*P* < 0.001) in younger participants in the top height quartile (−0.183 m/s) compared to those in the bottom quartile (−0.121 m/s), leading the GS difference between the two groups to be attenuated by 50% over the follow-up. The height-related difference in fast GS decline was not explained by time-dependent comorbidities or height shrinkage. Analyses for usual GS yielded consistent findings. The height-GS relation is more complex than previously thought, as the height related advantage in GS disappears as persons grow older due to faster decline in taller compared to shorter persons.

## Introduction

The ability to walk is essential for independent living and the speed of walking declines with age^1^. Gait speed is a simple and reproducible measure that can be easily implemented in clinical settings and carries remarkable prognostic information as slow gait speed is associated with unfavorable outcomes, including disability^2^, dementia^3^, and death^4^.

Height is one of the many factors that influences gait speed. The health advantages of greater height have long attracted scientific interest. In humans, taller stature is associated with lower risk of disease, cardiovascular disease in particular^5,6^, although recent studies suggest an association with some cancers^7^. Taller persons walk faster^8^, with longer legs playing a role. When the purpose is to identify abnormal gait speed values, it has been suggested that they should be scaled to body size to reduce inter-subject variability^9^. However, although taller stature is associated with faster gait speed, the extent to which this advantage persists into old age or is amplified or attenuated at older ages is not known.

In this paper, our research question is whether the gait speed advantage of taller stature is lost with age. To address this question, we investigated to which extent the association between taller stature and faster gait speed persists into old age. Hence, we used data from up to five gait speed assessments over 11 years in the French City (3 C)-Dijon cohort study to examine, in adults aged 65–85 years, whether the cross-sectional association with height varied with age and whether height influenced change in gait speed over the follow-up.

## Methods

### Study population

The 3 C study recruited community-dwelling older subjects ≥65 years from electoral rolls in three French cities^10^. The present study is based on data from the city of Dijon (n = 4,931) which included a specific assessment of motor function in participants aged 65–85 years. Participants ≤85 years were invited to the study centre in 1999–2001 and after two (wave 1/2001–02), four (wave 2/2003–04), seven (wave 4/2006–07), nine (wave 5/2008–09), and 11 years (wave 6/2010–12). Participants >85 years were assessed at home throughout the follow-up; from wave 2 onwards, those ≤85 years were also offered the option of a home assessment, and gait speed was assessed at home only at wave 6. Wave 3 (2005–06) included only a self-administered questionnaire.

The study protocol was approved by the ethical committee of the Kremlin-Bicêtre University-Hospital (France); all methods were performed in accordance with the declaration of Helsinki and all participants gave written informed consent.

### Outcome measure: gait speed

Gait speed was measured at the study centre using two photoelectric cells (6 meters [m] apart) connected to a chronometer. Participants were asked to walk at ‘usual’ and then ‘fast’ (without running) speed; they had three meters before the start line where the first photoelectric cell was placed to get their rhythm. For participants seen at home at wave 6 (n = 186, 16.7%), we used portable photoelectric cells (Racetime2 kit light radio, MicroGate®) with the same protocol, over 6 m in most instances (85%) or shorter distances (3.5–5.9 m) otherwise.

Speed of walking (m/s) was computed as distance (m) divided by time taken to cover the distance (seconds). Short-term reproducibility was assessed by repeating the assessment within 5 minutes in a random sample of 51 participants (9 men < 80 y; 10 men ≥ 80 y; 13 women < 80 y; 19 women ≥ 80 y); their height was comparable to the height of participants from the whole sample of similar age and sex. Intraclass correlation coefficients (Standard Error, SE) were: usual gait speed, 0.84 (0.02); fast gait speed, 0.92 (0.02)^11^. Given greater annual decline (0.019 vs. 0.007 m/s) and inter-individual variability (random slope variability, 0.025, SE = 0.002, vs. 0.011, SE = 0.001) for fast compared to usual gait speed, we used fast gait speed in the analysis as the main outcome and report results for usual gait speed in sensitivity analyses.

### Exposure variable: height

At baseline, height was measured to the nearest centimeter during clinical examinations using a stadiometer, while participants stood completely erect with the head in the Frankfort plane; height was self-reported for a small proportion of participants (1% of those included in the analyses). Height was measured again at wave 4 in 89% of the participants with a gait measure at that wave.

### Covariates

We assessed a wide range of covariates to describe the participants characteristics, including several that have been previously associated with either height or gait speed, and to perform multivariable analyses.

Weight was measured to the nearest kilogram during clinical examinations (or self-reported at all waves otherwise); measures were obtained for 99% of participants at baseline, 0% at waves 1 and 2, 89% at wave 4, 92% at wave 5, and 87% at wave 6. BMI (kg/m²) was calculated as weight divided by height squared; baseline height was used to compute BMI at baseline and waves 1–2, and height from wave 4 for BMI at waves 4–6. Socio-demographic covariates were drawn from the study baseline and included: age, sex, and education (low, no education/primary school; intermediate, secondary school; high, high-school/university degree). Additional covariates were assessed at baseline and at each wave of data collection. Cognition was assessed using the mini-mental state examination (MMSE), categorized in tertiles for the analyses. Depressive symptoms were evaluated with the Centre for Epidemiological Studies-Depression scale (CES-D)^12^; we used a cut-off of 16 to define the presence of depressive symptoms. History of bone fracture and falls was assessed over the two years preceding each visit. Low level of physical activity was defined as walking less than one hour per day and exercising less than once a week (assessed at baseline and waves 5 and 6; we used physical activity at baseline to impute values at waves 1 and 2, and physical activity at wave 5 to impute values at wave 4). History of knee or hip replacement for osteoarthritis, self-reported diabetes, dyspnea (New York Heart Association classification), osteoporosis, regular use of non-steroidal anti-inflammatory drugs (NSAIDs) for joint pain, use of psychotropic drugs, cardiovascular disease (non-disabling stroke, coronary heart disease (CHD), lower-limb arteritis), hypertension (systolic blood pressure ≥140 mm Hg or diastolic blood pressure ≥90 mm Hg or antihypertensive medication), and lipid lowering drugs as a surrogate for hypercholesterolemia were reported by participants. Incident stroke and CHD events were validated by expert committees based on medical records^13^. Cardiovascular disease was defined as having a history of stroke, CHD, or lower-limb arteritis.

#### Exclusion criteria

The following covariates were assessed at baseline and over the follow-up in order to exclude from the analyses participants with specific conditions that represent important causes of gait impairment. History of Parkinson’s disease and hip fracture in the previous two years were reported by participants. Disabling stroke was defined as a Rankin scale ≥3 (moderate, moderately severe, severe) or disability for mobility (Rosow and Breslau scale), ADL (Katz scale), or IADL (Lawton-Brody scale)^2^. The diagnosis of dementia at baseline and subsequent waves of data collection was established according to a standardized 3-step procedure^10,14^. First, trained neuropsychologists administered a battery of neuropsychological tests assessing memory, attention, language, and visuospatial abilities to all participants. Second, a neurologist examined all participants suspected of having dementia based on their neuropsychological evaluation (using age- and education-specific cut-offs for the MMSE, Benton Visual Retention Test, and Isaac Set Test). Third, a committee of expert neurologists reviewed all potential cases of dementia, and reached a consensus diagnosis based on standard criteria^15,16^.

### Statistical analysis

Participants with missing data on covariates (height, education) or conditions that represent important causes of gait impairment at study baseline or over the follow-up (Parkinson’s disease, dementia, hip fracture in the previous two years, disabling stroke) were excluded.

Participants’ characteristics were described using analysis of covariance. Height was categorized using sex-specific quartiles; *P*-values for trend were computed using orthogonal contrasts. We also ran sensitivity analyses using continuous height. BMI was categorized as (WHO classification): underweight (<18.5 kg/m^2^), normal (18.5–24.9 kg/m^2^), overweight (25.0–29.9 kg/m^2^), obese (≥30 kg/m^2^); as few participants (<2%) were underweight, we combined them with the normal group after ensuring that this did not affect our findings.

#### Cross-sectional analysis

The cross-sectional association between height and baseline gait speed was examined using linear regression. Model 1 included height, baseline age (centered at 65 years, divided by 10), and major gait speed correlates: sex, BMI, education. Model 2 was further adjusted for MMSE, cardiovascular disease, hypertension, hypercholesterolemia (all associated with both gait speed and height; Table 1). In order to examine whether the cross-sectional association changed with age, we included a height × age interaction term in the model. The corresponding equation is shown as supplementary methods.

**Table 1:** Participants’ Characteristics at Baseline (1999–2001).

Characteristics			Overall (N = 4011)		Fast gait speed^a^ (m/s) (SE) (N = 3,707)	Height^a^ (cm) (SE) (N = 4,011)
			Mean (SD)	No. (%)		
Age (years)			73.4 (4.6)	—	—	—
	<70			1162 (29.0)	1.672 (0.008)	164.6 (0.2)
	70–74			1117 (27.8)	1.571 (0.009)	163.4 (0.2)
	74–78			1049 (26.2)	1.509 (0.009)	162.8 (0.2)
	≥78			683 (17.0)	1.422 (0.011)^b,c^	161.5 (0.2)^b,c^
Sex	Men			1540 (38.4)	1.663 (0.007)	169.5 (0.2)
	Women			2471 (61.6)	1.425 (0.006)^b^	156.7 (0.1)^b^
Education	No education/primary school			1390 (34.7)	1.474 (0.008)	161.9 (0.2)
	Secondary school			1286 (32.1)	1.530 (0.008)	163.0 (0.2)
	High-school/university degree			1335 (33.3)	1.621 (0.008)^b,c^	164.3 (0.2)^b,c^
Height (cm)	Men	Women		161.8 (8.8)	—	—
	Q1, <165	Q1, <153		894 (22.3)	1.500 (0.009)	—
	Q2, 165–170	Q2, 153–157		1031 (25.7)	1.523 (0.009)	—
	Q3, 170–174	Q3, 157–161		1016 (25.3)	1.561 (0.009)	—
	Q4, ≥174	Q4, ≥161		1070 (26.7)	1.588 (0.009)^b,c^	—
BMI (kg/m²)			25.7 (4.0)	—	—	—
	Normal (<25)			1877 (46.8)	1.599 (0.007)	163.6 (0.1)
	Overweight (25–29.9)			1587 (39.6)	1.524 (0.007)	162.8 (0.2)
	Obese (≥30)			547 (13.6)	1.413 (0.012)^b,c^	162.2 (0.3)^b,c^
MMSE score	<27			966 (24.1)	1.487 (0.009)	162.3 (0.2)
	27–28			706 (17.6)	1.519 (0.011)	163.0 (0.2)
	≥28			2339 (58.3)	1.575 (0.006)^b,c^	163.4 (0.1)^b,c^
Depressive symptoms	Yes			893 (22.3)	1.467 (0.010)	163.0 (0.2)
	No			3113 (77.7)	1.564 (0.005)^b^	163.1 (0.1)
Bone fracture	Yes			273 (6.8)	1.521 (0.017)	163.4 (0.4)
	No			3738 (93.2)	1.545 (0.005)	163.1 (0.1)
Falls	Yes			223 (5.6)	1.463 (0.019)	162.9 (0.4)
	No			3787 (94.4)	1.548 (0.005)^b^	163.1 (0.1)
Physical activity	Low			943 (23.9)	1.490 (0.009)	163.0 (0.2)
	High			3002 (76.1)	1.562 (0.005)^b^	163.1 (0.1)
Diabetes	Yes			299 (7.5)	1.484 (0.016)	163.3 (0.4)
	No			3712 (92.5)	1.549 (0.005)^b^	163.1 (0.1)
Dyspnea	Yes			528 (13.2)	1.434 (0.012)	162.9 (0.3)
	No			3483 (86.8)	1.561 (0.005)^b^	163.1 (0.1)
NSAIDs for joint pain	Yes			605 (15.1)	1.462 (0.012)	163.1 (0.3)
	No			3398 (84.9)	1.558 (0.005)^b^	163.1 (0.1)
Knee/hip replacement for osteoarthritis	Yes			175 (4.4)	1.451 (0.021)	163.5 (0.5)
	No			3836 (95.6)	1.548 (0.005)^b^	163.1 (0.1)
Osteoporosis	Yes			826 (20.6)	1.538 (0.011)	163.2 (0.2)
	No			3179 (79.4)	1.545 (0.005)	163.0 (0.1)
Psychotropic drugs	Yes			1004 (25.0)	1.481 (0.009)	162.8 (0.2)
	No			3007 (75.0)	1.564 (0.005)^b^	163.2 (0.1)
Cardiovascular disease^d^	Yes			613 (15.3)	1.482 (0.011)	162.7 (0.2)
	No			3398 (84.7)	1.557 (0.005)^b^	163.2 (0.1)
Hypertension	Yes			3168 (79.0)	1.531 (0.005)	162.9 (0.1)
	No			843 (21.0)	1.597 (0.010)^b^	163.7 (0.2)^b^
Hypercholesterolemia	Yes			1341 (33.4)	1.526 (0.008)	162.6 (0.2)
	No			2670 (66.6)	1.553 (0.006)^b^	163.3 (0.1)^b^

Q1 (shortest)-Q4 (tallest), sex-specific quartiles of height; BMI, body mass index; MMSE, mini-mental state examination; NSAIDs, non-steroidal anti-inflammatory drugs; SD, standard deviation; SE, standard error.

^a^Age/sex-adjusted means and standard errors (SE). Analyses based on 3707 participants with a fast gait speed measure at baseline.

^b^Age- and sex-adjusted P-value < 0.001.

^c^Age- and sex-adjusted P-value for trend.

^d^Stroke, coronary heart disease, lower-limb arteritis.

#### Longitudinal analysis

The association between baseline height and change in gait speed over the follow-up was assessed using a linear mixed model with the intercept and slope (time in years, divided by 10, to yield 10 year change in gait speed) as random effects. We examined individual trajectories that showed a fairly linear decline in gait speed over time (Supplementary Figure 1); we also checked that adding a quadratic term for time did not improve the model’s fit (p = 0.19). Model 1 included height, baseline age (centered at 65 years, divided by 10), the significant height × age interaction, sex, time-dependent BMI, education, time, and significant interactions of time with height and age. Model 2 was additionally adjusted for time-dependent covariates associated with height (MMSE, bone fracture, knee/hip replacement for osteoarthritis, hypertension; Supplementary Table 1). The corresponding equations are shown as supplementary methods.

#### Missing data

Measures of gait speed over the follow-up were missing due to death, incident causes of gait impairment, being assessed at home (gait speed was assessed at home at wave 6 only), and general non-response. To examine the influence of missing data, we first examined the association between height and non-response using a proportional Cox hazards model. We then used a joint modeling approach to model simultaneously change in gait speed using a linear mixed model and time to drop-out or death with a survival model^17^.

Participants ≤85 y in whom gait speed was not measured (non-response, home exams, incident causes of gait impairment) were assumed to have dropped out. The joint model links the two sub-models through shared random effects, allowing dependency between the longitudinal process of change in gait speed and time to drop-out or death. This approach corrects longitudinal estimates (change in gait speed) by taking drop-out and death into account. The survival model included covariates associated with the risk of drop-out or death (sex, age, BMI). This approach was implemented through the *stjm* command in Stata 12.1 (College Station, TX: StataCorp LP).

#### Sensitivity analyses

We carried several sets of sensitivity analyses to check the robustness of our findings. We included height used as a continuous measure rather than in categories assuming a linear relation between gait speed and height. We assessed whether shrinkage (measured by the difference of gait speed between two waves (baseline, wave 4) may account for our findings by including height as a time dependent variable. We also performed analyses using usual rather than fast gait. We assessed whether self-reported height in some participants could have influenced our findings, both for cross-sectional and longitudinal analyses, by excluding participants who self-reported height or adjusting the analyses by including an indicator for self-reported height. We followed the same approach to examine whether home measures of gait speed at wave 6 in some participants could have influenced our findings.

Analyses were performed with SAS 9.3 (SAS Institute Inc, Cary, North Carolina). *P* values are 2 sided and those ≤0.05 considered statistically significant.

## Results

Participants older than 85 years at baseline (n = 239) and those with conditions that cause gait impairment (n = 171) or missing data on covariates (n = 13) were excluded. Of 4,508 participants eligible for the analysis, 497 did not have a fast gait speed measure over the follow-up; they were older (77.4 vs. 73.4 years, *P* < 0.001) but not different for height (161.9 cm,) than those with at least one measure (161.8 cm, age-adjusted *P* = 0.72). Of 4,011 subjects available for analysis, 664 (16.6%) had five fast gait speed assessments, 575 (14.3%) four, 569 (14.2%) three, 898 (22.4%) two, and 1,305 (32.5%) one.

Table 1 presents participants’ characteristics at study baseline. Men walked faster (mean fast gait speed [SD]: 1.679 [0.303] m/s) than women (1.438 [0.272] m/s), and were also taller (169.7 [6.4] vs. 156.8 [6.0] cm). Older, less educated, and obese participants walked slower and were shorter. Participants with a worse health profile walked slower. Shorter participants had lower MMSE scores than taller participants, and a more frequent history of hypertension, hypercholesterolemia, and cardiovascular disease. Supplementary Table 1 shows the association between height and time-dependent characteristics over the follow-up; taller participants maintained higher MMSE scores, and had an increased risk of bone fracture and knee/hip replacement for osteoarthritis, and a lower prevalence of hypertension.

Table 2 shows the baseline cross-sectional association between height (sex-specific quartiles) and fast gait speed; men and women were combined in the analysis as there were no sex-related differences in this association (P_Interaction_ = 0.72). Persons of short stature at age 65 years walked slower; this association remained unchanged after adjustment for covariates (model 2). However, the significant height × age interaction (*P* = 0.02) indicated that height related differences in fast gait speed were attenuated in older participants. The difference in fast gait speed between those in bottom and top quartiles of height at 65 years was 0.100 m/s (*P* < 0.001), 0.041 m/s (*P* = 0.002) in those aged 75 years, and 0.012 m/s (*P* = 0.57) in those aged 80 years.

**Table 2:** Cross-Sectional Association of Height With Fast Gait Speed at Study Baseline (1999–2001) (N = 3,707).

Characteristics	Model 1^a^			Model 2^b^		
	Beta	95% CI	*P*	Beta	95% CI	*P*
Intercept (m/s)^c^	1.540	1.498, 1.582	<0.001	1.581	1.536, 1.626	<0.001
Age (10 years, centered at 65 years)	−0.158	−0.195, −0.122	<0.001	−1.470	−0.183, −0.111	<0.001
Height						
Q1	Reference			Reference		
Q2	0.034	−0.018, 0.087	0.20	0.034	−0.019, 0.086	0.21
Q3	0.073	0.022, 0.124	0.01	0.068	0.017, 0.119	0.01
Q4	0.100	0.050, 0.150	<0.001	0.096	0.046, 0.146	<0.001
		Trend	<0.001		Trend	<0.001
Height × Age (10 years, centered at 65 years)						
Q1	Reference			Reference		
Q2	−0.030	−0.080, 0.020	0.24	−0.031	−0.081, 0.019	0.22
Q3	−0.047	−0.098, 0.004	0.07	−0.044	−0.095, 0.006	0.09
Q4	−0.059	−0.110, −0.007	0.03	−0.057	−0.108, −0.006	0.03
		Trend	0.02		Trend	0.03

Beta, regression coefficients; CI, confidence interval; Q1 (shortest)-Q4 (tallest), sex-specific quartiles of height; BMI, body mass index; MMSE, mini-mental state examination.

Please see the supplementary methods for the equations corresponding to the models presented in the table.

^a^Adjusted for sex (reference, women), education (reference, low education), baseline BMI (reference, <25 kg/m^2^).

^b^Model 1 +MMSE, history of cardiovascular disease (stroke, coronary heart disease, lower-limb arteritis), hypertension, hypercholesterolemia.

^c^The intercept (model 1) corresponds to the average fast gait speed for women aged 65 years at baseline, with normal BMI, low education, and in the lower quartile of height. In model 2, the reference group for covariates were: highest tertile of MMSE, no cardiovascular disease, no history of hypertension, no hypercholesterolemia.

**Figure 1:**
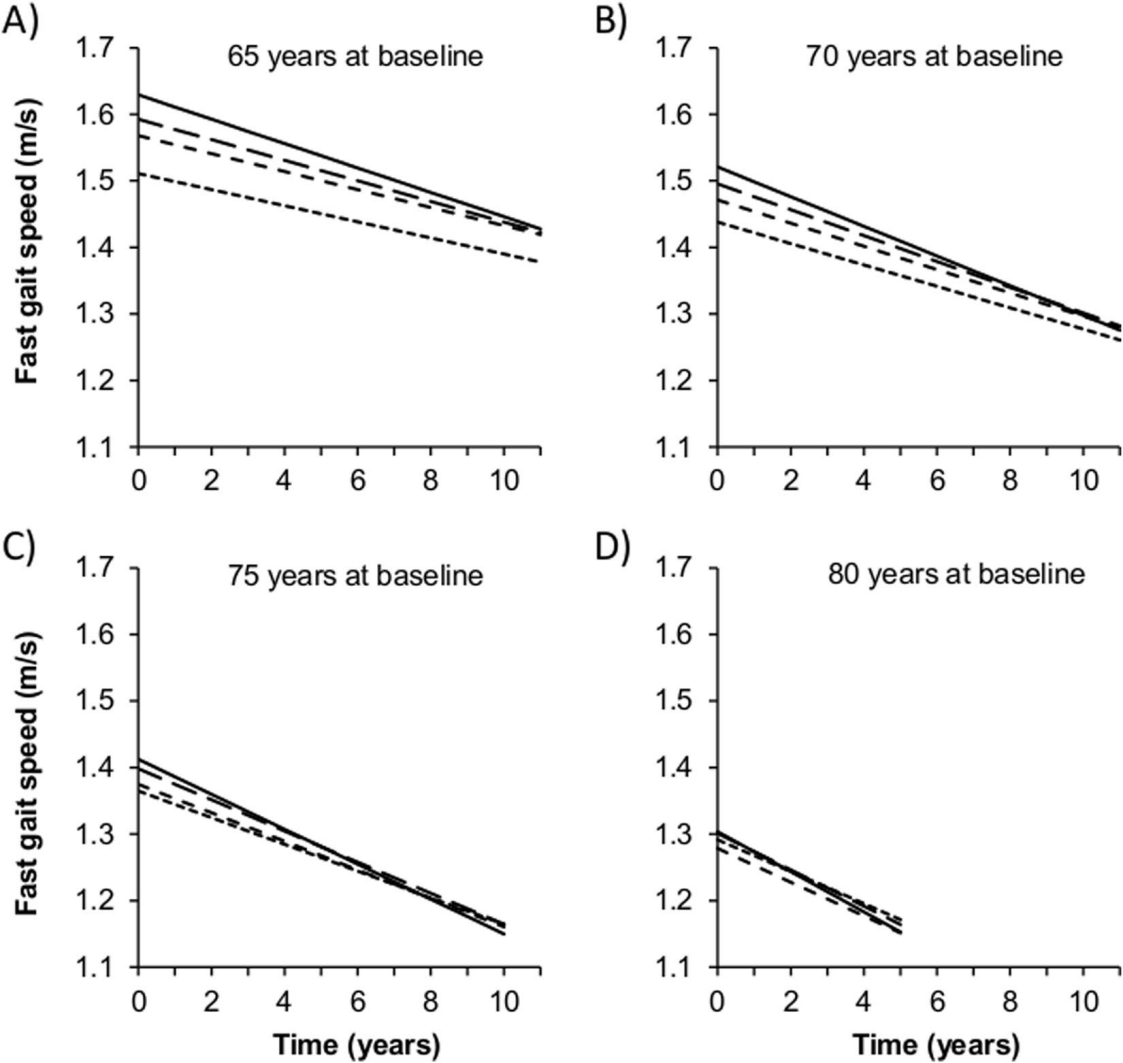
Predicted Trajectories of Fast Gait Speed According to Quartiles of Height in Women Aged 65 (**A**), 70 (**B**), 75 (**C**), and 80 (**D**) Years at Study Baseline. Fast gait speed was modelled using a linear mixed model including a random intercept and slope (results are shown in Table 3 and the corresponding equations as supplementary methods). Quartiles of height: short dashed line, Q1 (shorter); intermediate dashed line, Q2; long dashed line, Q3; solid line, Q4 (taller).

Table 3 and Fig. 1 present associations of baseline height with change in fast gait speed over the follow-up. Decline in fast gait speed was more pronounced in older (P_Age×Time_ < 0.001) and taller (P_Trend Height×Time_ < 0.001) participants. In participants 65 years at baseline, gait speed decline was 51% greater for those in the top quartile of height (10 year change, −0.183 m/s) compared to those in the bottom quartile (10 year change, −0.121 m/s; difference, −0.062 m/s, *P* < 0.001); this association between the top quartile of height and change in gait speed is equivalent to the effect of 7.8 ([10/0.079] × 0.062) years of age on gait speed decline. As shown in Fig. 1, the mean difference in fast gait speed between the top and bottom quartiles of height in women 65 years at baseline (0.118 m/s, *P* < 0.001) was considerably attenuated after a 10 year follow-up (0.056 m/s, *P* = 0.02). In participants ≥75 years at baseline, fast gait speed trajectories in tallest and shortest participants converged over the 10 year follow-up. The time × height interaction did not depend on sex (*P* = 0.33) or age at baseline (*P* = 0.15). The longitudinal association between height and fast gait speed remained unchanged after adjustment for covariates (model 2).

**Table 3:** Association Between Height at Baseline and Change in Fast Gait Speed Over the Follow-up (N = 4,011).

Characteristics	Model 1^a^			Model 2^b^		
	Beta	95% CI	*P*	Beta	95% CI	*P*
10 year change in fast walking speed (m/s)	−0.121	−0.154, −0.089	<0.001	−0.112	−0.145, −0.080	<0.001
Baseline age (10 years) × 10 year change in fast gait speed (m/s)	−0.079	−0.109, −0.049	<0.001	−0.076	−0.106, −0.046	<0.001
Baseline height × 10 year change in fast gait speed (m/s)						
Q1	Reference			Reference		
Q2	−0.014	−0.047, 0.019	0.41	−0.014	−0.046, 0.019	0.42
Q3	−0.033	−0.066, −0.001	0.044	−0.032	−0.065, 0.000	0.050
Q4	−0.062	−0.094, −0.030	<0.001	−0.061	−0.093, −0.029	<0.001
		Trend	<0.001		Trend	<0.001

Beta, regression coefficients; CI, confidence interval; Q1 (shortest)-Q4 (tallest), sex-specific quartiles of height; BMI, body mass index; MMSE, mini-mental state examination.

Please see the supplementary methods for the equations corresponding to the models presented in the table.

^a^Adjusted for sex (reference, women), education (reference, low education), time-dependent BMI (reference, <25 kg/m^2^), baseline height (reference, Q1), baseline age (centered at 65 years), baseline height×baseline age.

^b^Model 1 +time-dependent covariates: MMSE (reference, highest tertile), bone fracture (reference, no), knee/hip replacement for osteoarthritis (reference, no), hypertension (reference, no).

Sensitivity analyses supported our findings. Height used as a continuous measure yielded results consistent with our main analyses (Supplementary Table 2); the effect of 10 cm of height is comparable to the effect of 4.2 years of age ([10/0.081] × 0.034) on gait speed decline. Based on repeat assessments of height (baseline, wave 4) on 1,520 participants, 10 year change in height between baseline and wave 4 was −2.0 cm (95% CI: −2.2, −1.9, *P* < 0.001). However, shrinkage is an unlikely explanation for our findings, as it was similar across quartiles of baseline height (in cm: −2.0, −2.0, −2.1, −2.1; *P* = 0.31). In addition, including height as a time-dependent variable in this subsample yielded similar findings (Supplementary Table 2). Height was not associated with non-response over the follow-up (age and sex-adjusted relative risk of non-response per 10 cm increase in height = 0.97; 95% CI: 0.89, 1.06; *P* = 0.51). Taking into account missing data through joint modeling confirmed our main findings (Supplementary Table 2). Analyses using usual rather than fast gait speed also yielded similar findings (Supplementary Table 3, Supplementary Figure 2). There was some evidence that the relative difference in decline across quartiles of height was even more pronounced for usual gait speed: participants in the top quartile experienced 158% faster decline than those in the bottom quartile. Similar conclusions were reached when taking into account participants with home measures of gait speed at wave 6 (Supplementary Table 4) or a small proportion (1%) who self-reported height (data not shown).

## Discussion

Based on a large cohort of older adults followed for 11 years with up to five assessments of gait speed, we examined whether the cross-sectional association with height varied with age and whether height influenced change in gait speed over the follow-up. Our main finding is that taller stature is associated with considerably faster gait speed in early old age but not at older ages. In other words, the cross-sectional association of height with gait speed decreased between 65 y and 80 y. Our longitudinal analyses show that this resulted from a steeper gait speed decline over time in taller participants.

Taller stature is associated with a number of health advantages, including better cognition^18^, lower risk of heart failure^19^, coronary artery disease^5,6^, stroke, respiratory disease, and death^20^. It is generally thought that health, growth, nutrition, and social environment in early life play an important role in explaining this protective effect. There is also evidence of greater risk of adverse events such as atrial fibrillation^21^, hip/wrist fracture^22^, hip/knee replacement^23^, low back pain^24^, and some cancers^7^ in taller individuals.

Taller individuals walk faster than their shorter counterparts^8^, and there are multiple mechanisms underlying this association. Taller persons tend to be more educated, have lower BMI, better cardiovascular risk profile, and higher cognitive function, all of which have been associated with faster gait speed^25^. However, in our study adjusting for these confounders had little impact on the association between height and gait speed. Thus, it is likely that mechanical factors, such as greater stride length of taller persons^26^, play an important role. The higher stride frequency of shorter persons does not compensate for shorter stride, leading to slower gait speed compared to their taller counterparts^27^.

To our knowledge, no other study has examined the impact of height on age-related change in gait speed. Therefore, we can only speculate about the reasons behind our findings. As reported by others, taller participants in our study had more bone fractures^22^ and knee/hip replacement for osteoarthritis^23^ over the follow-up. However, adjustment for these factors had little impact on our results. The same was true for cognitive function. Shrinkage, i.e., height loss with ageing, was another factor we examined. It was modest in our study and consistent with previous estimates (2 cm/10 years), but it was not associated with baseline height; furthermore, adjustment for time-dependent height in participants with two measures of height (seven years apart) yielded similar results. Height loss is mainly due to compression of the discs between the vertebrae in the spine and fractures of the vertebrae due to osteoporosis^28^. Therefore, it is not unexpected that it did not have a strong impact on gait speed decline. However, height loss is more pronounced in case of osteoporosis and has been associated with an increased risk of hip fracture^29^; it can therefore serve as a surrogate for osteoporosis.

Gait patterns change with age: stride length shortens, stride frequency reduces, and gait becomes irregular, steps wider, and foot clearance more problematic^30^. Previous studies suggest that slowing of gait with ageing may be a way to compensate for greater energetic cost of walking. In other words, older adults slow down in order to minimize energy expenditure (‘economy of mobility’)^31,32^. Therefore, one possible hypothesis to explain our findings is that, as they age, individuals adopt a ‘basal’ slow gait speed to conserve energy, and that this ‘basal’ speed is independent of their height. Because taller stature is associated with greater energy expenditure in absolute terms^27^ adoption of a basal slow gait speed by taller people would lead to a steeper decline in gait speed because they initially walked faster and would allow them to walk at a ‘minimal’ pace consistent with independent living, while at the same time saving energy. Further research is needed to better understand the impact of height on gait speed and energy expenditure in older adults.

Our findings contribute to a better understanding of the individual characteristics that have an influence on decline of gait speed in elderly people and to make hypotheses regarding the physiology of gait during the ageing process. In addition, when the purpose is to identify abnormal gait speed values, some authors have suggested that they should be scaled to body size, height in particular, to reduce inter-subject variability^9^. The age-dependent association between height and gait speed implies that this may be less important in older subjects than in younger subjects; hence, gait speed norms in different age groups should take the age-dependent relation between height and gait speed into account.

The main limitation of our study is that gait speed was not measured at each wave for all participants because some died or dropped-out over the course of the follow-up, as in all longitudinal studies in older adults. However, height was not significantly associated with non-response. Furthermore, we used joint modeling to take missing values into account in order to assess the robustness of our findings. Second, height was not measured over the follow-up at each wave on all subjects. However, analyses based on a subgroup of participants in whom height was measured twice showed that shrinkage was small and independent of baseline height, making it an unlikely explanation for our findings. Third, although the correlation is not strong, increased height is associated with poorer balance^33^, and this association may contribute to our findings if it became more pronounced with increasing age; further studies with sensitive measures of balance will be necessary to test this hypothesis. Finally, our conclusions are limited to the 65–85 years age range. This study’s main strengths include its large size and long follow-up with up to five gait speed assessments. The main outcome is an objective and reproducible measure of motor performance. In addition, although the rate of decline was more pronounced for fast than usual gait speed, findings were consistent across the measures.

In conclusion, while it is well known that taller persons have longer legs and walk faster, we now provide robust evidence that this relation is more complex than previously thought and that this difference is completely attenuated by age 80 y.

## Electronic supplementary material

Supplementary material
