# Supplementary material for: The gait speed advantage of taller stature is lost with age
Source: Sci Rep. 2018 Jan 24;8:1485. doi: 10.1038/s41598-018-19882-1 (PMC5784135; doi:10.1038/s41598-018-19882-1)
Supplement: Supplementary file 1 — Supplementary material [file 41598_2018_19882_MOESM1_ESM.pdf]

The gait speed advantage of taller stature is lost with age

Alexis Elbaz, MD, PhD[1], Fanny Artaud, PhD[1], Aline Dugravot, MSc[1], Christophe Tzourio, MD, PhD[2,3], Archana Singh-Manoux, PhD[1,4]

1. Université Paris-Saclay, Univ. Paris-Sud, UVSQ, CESP, INSERM, Villejuif, France
2. INSERM U897, Neuroepidemiology team, Bordeaux, France
3. University of Bordeaux, Bordeaux, France
4. Department of Epidemiology and Public Health, University College London, UK

Supplementary material: supplementary methods, 4 tables, 2 figures.

Supplementary methods

Cross-sectional association of height with fast gait speed at baseline (1999-2001) (table 2)

We used linear regression to model gait speed at baseline as a function of height at baseline while adjusting for covariates (model 1):

$$Gait\ speed_i = \beta_0 + \beta_{Q2}Q2_{Height_i} + \beta_{Q3}Q3_{Height_i} + \beta_{Q4}Q4_{Height_i} + \beta_{Age}Age_i + \beta_{Q2\times Age}\left(Q2_{Height_i} \times Age_i\right) + \beta_{Q3\times Age}\left(Q3_{Height_i} \times Age_i\right) + \beta_{Q4\times Age}\left(Q4_{Height_i} \times Age_i\right) + \beta_{Sex}Sex_i + \sum_1^2 \beta_{Edu}\, Edu_i + \sum_1^2 \beta_{BMI}\, BMI_i + \varepsilon_i$$

where

- $Gait\ speed_i$ is fast gait speed at baseline of the ith participant (m/s),
- β_0 (intercept) is the average baseline fast gait speed in subjects with reference values for the covariates at baseline (women, 65 years, normal BMI, low education, Q1 of height),
- The other β coefficients are the regression coefficients associated with the covariates,
- $Height_i$ is the baseline height of the ith participant categorized in sex-specific quartiles ($Q1 - Q4_{Height_i}$, $Q1_{Height_i}$ is the reference),
- Age_i is age at baseline of the ith participant, centered at 65 years and divided by 10,
- $Q2 -\ Q4_{Height_i} \times Age_i$ are the three interactions between age and quartiles $Q2 - Q4$ of height,
- Sex_i is coded 0 for women (reference) and 1 for men,
- Edu_i is the education level of the ith participant in 3 categories (low, reference),
- BMI_i is the baseline body mass index of the ith participant in 3 categories (reference, <25 kg/m²),
- ε_i is the residual of the ith participant.

Model 2 is further adjusted for baseline MMSE, history of cardiovascular disease (stroke, coronary heart disease, lower-limb arteritis), hypertension, and hypercholesterolemia.

Association between height at baseline and change in fast gait speed over the follow-up (table 3)

We used a linear mixed model to model change in gait speed over time as a function of height at baseline while adjusting for covariates (model 1):

$$Gait\ speed_{ij} = \beta_0 + \beta_{Q2}Q2_{Height_i} + \beta_{Q3}Q3_{Height_i} + \beta_{Q4}Q4_{Height_i} + \beta_{Age}Age_i + \beta_{Q2\times Age}(Q2_{Height_i} \times Age_i) + \beta_{Q3\times Age}(Q3_{Height_i} \times Age_i) + \beta_{Q4\times Age}(Q4_{Height_i} \times Age_i) + \beta_{Sex}Sex_i + \sum_1^2 \beta_{Edu}\, Edu_i + \beta_{Time}Time_{ij} + \beta_{Q2\times Time}(Q2_{Height_i} \times Time_{ij}) + \beta_{Q3\times Time}(Q3_{Height_i} \times Time_{ij}) + \beta_{Q4\times Time}(Q4_{Height_i} \times Time_{ij}) + \beta_{Age\times Time}(Age_i \times Time_{ij}) + \sum_1^2 \beta_{BMI}\, BMI_{ij} + U_{0i} + U_{1j}Time_{ij} + \varepsilon_{ij}$$

where

- $Gait\ speed_{ij}$ is the fast gait speed of the ith participant at the jth visit (m/s),
- β_0 (intercept) is the average baseline fast gait speed in subjects with reference values for the covariates at baseline (women, 65 years at baseline, normal BMI, low education, Q1 of height),
- The other β coefficients are the regression coefficients associated with the covariates,
- $Height_i$ is the baseline height of the ith participant categorized in sex-specific quartiles ($Q1_{Height_i}$ to $Q4_{Height_i}$ $Q1_{Height_i}$ is the reference),
- Age_i is age at baseline of the ith participant, centered at 65 years and divided by 10,
- $Q2 - Q4_{Height_i} \times Age_i$ are the three interactions between age and quartiles $Q2 - Q4$ of height at baseline,
- Sex_i is coded 0 for women (reference) and 1 for men,
- Edu_i is the education level of the ith participant in 3 categories (low, reference),
- $Time_{ij}$ is time since baseline for the ith participant at the jth visit (in years, divided by 10), and β_{Time} is the average slope of change in gait speed over time in subjects with reference values for the covariates (women, 65 years at baseline, normal BMI, low education, Q1 of height),
- $Q2 - Q4_{Height_i} \times Time_{ij}$ are the three interactions between time since baseline and quartiles $Q2 - Q4$ of height,
- $Age_i \times Time_{ij}$ is the interaction between time since baseline and age at baseline,
- BMI_{ij} is the time-dependent body mass index of the ith participant at the jth visit in 3 categories (reference, <25 kg/m^2),
- U_{0i} is the random intercept and U_{1i} is the random slope of the ith participant,
- ε_{ij} is the residual of the ith participant at the jth visit.

Model 2 is further adjusted for time-dependent MMSE, history of cardiovascular disease (stroke, coronary heart disease, lower-limb arteritis), hypertension, hypercholesterolemia.

In supplementary table 2-A, height is modelled as a continuous variable, rather than in quartiles:

$$Gait\ speed_{ij} = \beta_0 + \beta_{Height}Height_i + \beta_{Age}Age_i + \beta_{Height\times Age}(Height_i \times Age_i) + \beta_{Sex}Sex_i + \sum_1^2 \beta_{Edu}\, Edu_i + \beta_{Time}Time_{ij} + \beta_{Height\times Time}\left(Height_i \times Time_{ij}\right) + \beta_{Age\times Time}\left(Age_i \times Time_{ij}\right) + \sum_1^2 \beta_{BMI}\, BMI_{ij} + U_{0i} + U_{1j}Time_{ij} + \varepsilon_{ij}$$

where $Height_i$ is continuous height at baseline, centered at 162 cm and divided by 10.

In supplementary table 2-B, height is modelled as a time-dependent variable, rather than a time-invariant variable fixed at baseline:

$$Gait\ speed_{ij} = \beta_0 + \beta_{Q2}Q2_{Height_{ij}} + \beta_{Q3}Q3_{Height_{ij}} + \beta_{Q4}Q4_{Height_{ij}} + \beta_{Age}Age_i + \beta_{Q2\times Age}\left(Q2_{Height_{ij}} \times Age_i\right) + \beta_{Q3\times Age}\left(Q3_{Height_{ij}} \times Age_i\right) + \beta_{Q4\times Age}\left(Q4_{Height_{ij}} \times Age_i\right) + \beta_{Sex}Sex_i + \sum_1^2 \beta_{Edu}\, Edu_i + \beta_{Time}Time_{ij} + \beta_{Q2\times Time}\left(Q2_{Height_{ij}} \times Time_{ij}\right) + \beta_{Q3\times Time}\left(Q3_{Height_{ij}} \times Time_{ij}\right) + \beta_{Q4\times Time}\left(Q4_{Height_{ij}} \times Time_{ij}\right) + \beta_{Age\times Time}\left(Age_i \times Time_{ij}\right) + \sum_1^2 \beta_{BMI}\, BMI_{ij} + U_{0i} + U_{1j}Time_{ij} + \varepsilon_{ij}$$

where $Height_{ij}$ is the time-dependent height of the ith participant at the jth visit.

Supplementary Table 1. Time dependent participants' characteristics and their relation with baseline height

Characteristics		No. (%)	Baseline mean height[a] (cm) (SE)	P^a
MMSE score[c]	< 27	1231 (30.7)	162.4 (0.2)	
	[27 ; 28[	944 (23.5)	162.9 (0.2)	
	≥ 28	1834 (45.7)	163.6 (0.1)	<0.001[b]
Depressive symptoms[d]	Yes	1275 (31.8)	163.1 (0.2)	
	No	2731 (68.2)	163.1 (0.1)	0.87
Bone fracture[d]	Yes	536 (13.4)	163.7 (0.3)	
	No	3475 (86.6)	163.0 (0.1)	0.01
Falls[d]	Yes	835 (20.8)	163.2 (0.2)	
	No	3175 (79.2)	163.1 (0.1)	0.60
Physical activity[d]	Low	1472 (37.3)	163.0 (0.2)	
	High	2473 (62.7)	163.1 (0.1)	0.71
Diabetes[d]	Yes	466 (11.6)	163.3 (0.3)	
	No	3545 (88.4)	163.0 (0.1)	0.39
Dyspnea[d]	Yes	869 (21.7)	163.2 (0.2)	
	No	3142 (78.3)	163.0 (0.1)	0.36
NSAIDs for joint pain[d]	Yes	869 (21.7)	163.2 (0.2)	
	No	3134 (78.3)	163.0 (0.1)	0.36
Knee/hip replacement	Yes	343 (8.6)	163.9 (0.3)	
for osteoarthritis[d]	No	3668 (91.4)	163.0 (0.1)	0.01
Osteoporosis[d]	Yes	1172 (29.3)	163.2 (0.2)	
	No	2833 (70.7)	163.0 (0.1)	0.56
Psychotropic drugs[d]	Yes	1411 (35.2)	163.0 (0.2)	
	No	2600 (64.8)	163.1 (0.1)	0.56
Cardiovascular disease[d,e]	Yes	831 (20.7)	162.9 (0.2)	
	No	3180 (79.3)	163.1 (0.1)	0.43
Hypertension[d]	Yes	3510 (87.5)	163.0 (0.1)	
	No	501 (12.5)	163.5 (0.3)	0.08
Hypercholesterolemia[d]	Yes	1752 (43.7)	162.9 (0.2)	
	No	2259 (56.3)	163.2 (0.1)	0.22

Abbreviations: SE, standard error; MMSE, mini-mental state examination; NSAID, non-steroidal anti-inflammatory drug.
[a] Age and sex adjusted means, standard errors (SE), and p-values are reported.
[b] *P* for trend.
[c] Mean of all measures taken during the follow-up.
[d] At least one report over follow-up.
[e] Stroke, coronary heart disease, lower-limb arteritis.

Supplementary Table 2. Association of height and decline in fast gait speed over the follow-up: sensitivity analyses

	Beta	95% CI	*P*
**A- Continuous height (N = 4,011)[a]**			
10 year change in fast gait speed (m/s)	-0.149	-0.171, -0.127	<0.001
Baseline age (10 years) × 10 year change in fast gait speed (m/s)	-0.081	-0.111, -0.051	<0.001
Baseline height (10 cm) × 10 year change in fast gait speed (m/s)	-0.034	-0.047, -0.022	<0.001
**B- Time-dependent height (N = 1,520)[b]**			
10 year change in fast gait speed (m/s)	-0.132	-0.170, -0.094	<0.001
Baseline age (10 years) × 10 year change in fast gait speed (m/s)	-0.099	-0.138, -0.059	<0.001
Time-dependent height × 10 year change in fast gait speed (m/s)			
Q1	Reference	--	--
Q2	0.010	-0.029, 0.048	0.63
Q3	-0.019	-0.058, 0.020	0.34
Q4	-0.049	-0.087, -0.011	0.010
		Trend	0.004
**C- Joint model of change in gait speed and time to drop-out (N = 4,011)[c]**			
10 year change in fast gait speed (m/s)	-0.125	-0.158, -0.092	<0.001
Baseline age (10 years) × 10 year change in fast gait speed (m/s)	-0.099	-0.130, -0.068	<0.001
Baseline height × 10 year change in fast gait speed (m/s)			
Q1	Reference		
Q2	-0.014	-0.046, 0.019	0.42
Q3	-0.034	-0.066, -0.012	0.042
Q4	-0.061	-0.093, -0.029	<0.001
		Trend	<0.001

Beta, regression coefficients; CI, confidence interval; Q1 (shortest)-Q4 (tallest), sex-specific quartiles of height; BMI, body mass index.
Please see the supplementary methods for the equations corresponding to the models presented in the table.
[a] Adjusted for sex (reference, women), education (reference, low education), time-dependent BMI (reference, normal), baseline height (centered at 162 cm), baseline age (years, centered at 65 years), baseline height × baseline age.
[b] Analyses based on 1,520 participants in whom height was measured both at baseline and wave 4. Adjusted for sex (reference, women), education (reference, low education), time-dependent BMI (reference, normal), time-dependent height (reference, first quartile), baseline age (years, centered at 65 years), time-dependent height × baseline age.
[c] Adjusted for sex (reference, women), education (reference, low education), time-dependent BMI (reference, normal), baseline height (reference, first quartile), baseline age (years, centered at 65 years), baseline height × baseline age.

Supplementary Table 3. Cross-sectional and longitudinal association of height with *usual gait speed*

	Beta	95% CI	*P*
**A- Cross-sectional association at baseline (N = 3,779)[a]**			
Intercept (m/s)	1.145	1.115, 1.174	<0.001
Age (years, centered at 65 years)	-0.126	-0.151, -0.101	<0.001
Height			
Q1	Reference		
Q2	0.004	-0.033, 0.040	0.82
Q3	0.030	-0.005, 0.066	0.097
Q4	0.074	0.039, 0.110	<0.001
		Trend	<0.001
Height × Age (10 years, centered at 65 years)			
Q1	Reference		
Q2	-0.003	-0.032, 0.038	0.88
Q3	-0.015	-0.050, 0.020	0.41
Q4	-0.055	-0.091, -0.020	0.002
		Trend	0.001
**B- Longitudinal association (N = 4,069)[b]**			
10 year change in usual gait speed (m/s)	-0.025	-0.049, -0.000	0.048
Baseline age (10 years) × 10 year change in usual gait speed (m/s)	-0.054	-0.077, -0.032	<0.001
Baseline height × 10 year change in usual gait speed (m/s)			
Q1	Reference		
Q2	-0.010	-0.034, 0.015	0.43
Q3	-0.024	-0.049, 0.000	0.050
Q4	-0.039	-0.062, -0.015	0.0014
		Trend	<0.001

Beta, regression coefficients; CI, confidence interval; Q1 (shortest)-Q4 (tallest), sex-specific quartiles of height; BMI, body mass index.

[a] Analyses based on 3,779 participants in whom usual gait speed (m/s) was measured at study baseline. Adjusted for sex (reference, women), education (reference, low education), baseline BMI (reference, normal). The intercept corresponds to the average usual gait speed for women aged 65 years at baseline, with normal BMI, low education, and in the lower quartile of height.

[b] Analyses based on 4,069 participants in whom usual gait speed (m/s) was measured at least once. Adjusted for sex (reference, women), education (reference, low education), time-dependent BMI (reference, normal), time-dependent BMI × sex, baseline height (reference, first quartile), baseline age (years, centered at 65 years), baseline height × baseline age.

Supplementary Table 4. Longitudinal association of height with fast gait speed taking into account home measures at wave 6.

	Beta[a]	95% CI[a]	*P*[a]
**A- Exclusion of home measures of fast gait speed at wave 6 for 186 participants**			
10 year change in usual gait speed (m/s)	-0.115	-0.148, -0.082	<0.001
Baseline age (10 years) × 10 year change in usual gait speed (m/s)	-0.075	-0.105, -0.045	<0.001
Baseline height × 10 year change in usual gait speed (m/s)			
Q1	Reference		
Q2	-0.016	-0.049, 0.017	0.33
Q3	-0.035	-0.067, -0.002	0.039
Q4	-0.064	-0.096, -0.032	<0.001
		Trend	<0.001
**B- Adjusted for home measures of fast gait speed at wave 6[b]**			
10 year change in usual gait speed (m/s)	-0.111	-0.144, -0.079	<.001
Baseline age (10 years) × 10 year change in usual gait speed (m/s)	-0.081	-0.111, -0.051	<.001
Baseline height × 10 year change in usual gait speed (m/s)			
Q1	Reference		
Q2	-0.015	-0.048, 0.017	0.36
Q3	-0.035	-0.067, -0.002	0.036
Q4	-0.064	-0.096, -0.033	<0.001
		Trend	<0.001

Beta, regression coefficients; CI, confidence interval; Q1 (shortest)-Q4 (tallest), sex-specific quartiles of height; BMI, body mass index; MMSE, mini-mental state examination.
[a]Adjusted for sex (reference, women), education (reference, low education), time-dependent BMI (reference, <25 kg/m^2), baseline height (reference, Q1), baseline age (centered at 65 years), baseline height×baseline age.
[b]We included an indicator to identify home measures of gait speed at wave 6.

Supplementary Figure 1. Individual trajectories of fast gait speed over the follow-up

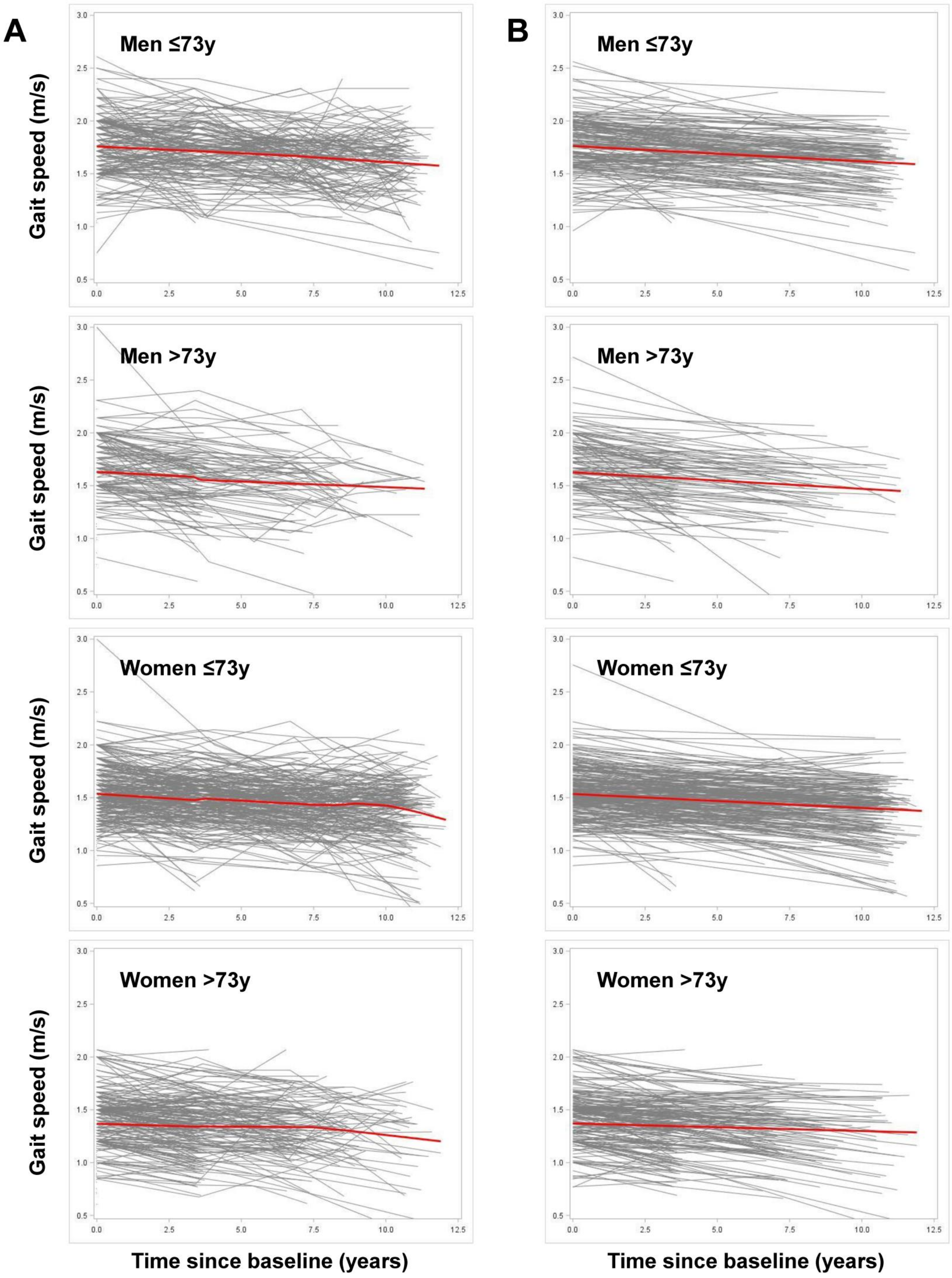

Individual trajectories were studied in a proportional random sample of 1200 subjects after stratification by sex and median age. Panel A shows individual trajectories of gait speed in grey and a fairly linear decline. The red line corresponds to a smoothed summary curve and also shows a fairly linear decline. Panel B shows OLS (ordinary least square regression) individual trajectories in grey that assume a linear decline and the corresponding pooled summary curve that is very similar to the pooled curve from Panel A.

Men ≤73y, n=238; men >73y, 222; women ≤73y, 363, women >73y, 377.

Supplementary Figure 2. Predicted trajectories of *usual gait speed* over the follow-up according to quartiles of baseline height in women aged 65 years old at baseline

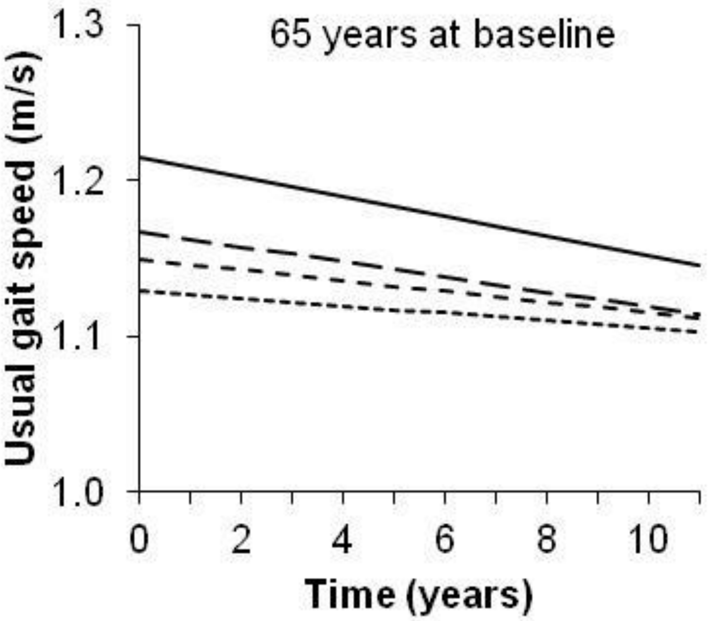

Usual gait speed was modelled using a linear mixed model including a random intercept and slope. The estimates from the model used to draw this figure are presented in supplementary table 3. Quartiles of height: short dashed line, Q1 (shortest); intermediate dashed line, Q2; long dashed line, Q3; solid line, Q4 (tallest).
